# Probing Subunit-Subunit Interactions in the Yeast Vacuolar ATPase by Peptide Arrays

**DOI:** 10.1371/journal.pone.0046960

**Published:** 2012-10-12

**Authors:** Lee S. Parsons, Stephan Wilkens

**Affiliations:** Department of Biochemistry and Molecular Biology, SUNY Upstate Medical University, Syracuse, New York, United States of America; The Scripps Research Institute, United States of America

## Abstract

**Background:**

Vacuolar (H^+^)-ATPase (V-ATPase; V_1_V_o_-ATPase) is a large multisubunit enzyme complex found in the endomembrane system of all eukaryotic cells where its proton pumping action serves to acidify subcellular organelles. In the plasma membrane of certain specialized tissues, V-ATPase functions to pump protons from the cytoplasm into the extracellular space. The activity of the V-ATPase is regulated by a reversible dissociation mechanism that involves breaking and re-forming of protein-protein interactions in the V_1_-ATPase - V_o_-proton channel interface. The mechanism responsible for regulated V-ATPase dissociation is poorly understood, largely due to a lack of detailed knowledge of the molecular interactions that are responsible for the structural and functional link between the soluble ATPase and membrane bound proton channel domains.

**Methodology/Principal Findings:**

To gain insight into where some of the stator subunits of the V-ATPase associate with each other, we have developed peptide arrays from the primary sequences of V-ATPase subunits. By probing the peptide arrays with individually expressed V-ATPase subunits, we have identified several key interactions involving stator subunits E, G, C, H and the N-terminal domain of the membrane bound *a* subunit.

**Conclusions:**

The subunit-peptide interactions identified from the peptide arrays complement low resolution structural models of the eukaryotic vacuolar ATPase obtained from transmission electron microscopy. The subunit-subunit interaction data are discussed in context of our current model of reversible enzyme dissociation.

## Introduction

The vacuolar ATPase (V-ATPase; V_1_V_o_-ATPase) is a large multisubunit enzyme complex that is found in the in the endomembrane system of all eukaryotic organisms where its ATP hydrolysis driven proton pumping function serves to acidify the lumen of intracellular organelles [1]–[4]. In polarized cells of animals, V-ATPase function in the plasma membrane leads to acidification of the extracellular milieu, a process essential for bone remodeling [5], urine acidification [6] and pH homeostasis [7]. Aberrant V-ATPase activity has been linked to a number of human diseases including diabetes [8], osteoporosis [9], renal tubular acidosis [10], infertility [11], and sensorineural deafness [12]. Furthermore, V-ATPase mediated acidification of compartments such as endosomes and phagosomes plays an essential role in dendritic cell maturation [13], viral entry [14] and antigen processing [15]. Due to its fundamental role in a large number of human diseases, great effort is spent on identifying potential drug molecules that may serve to modulate aberrant V-ATPase activity [16]–[18].

**Figure 1:**
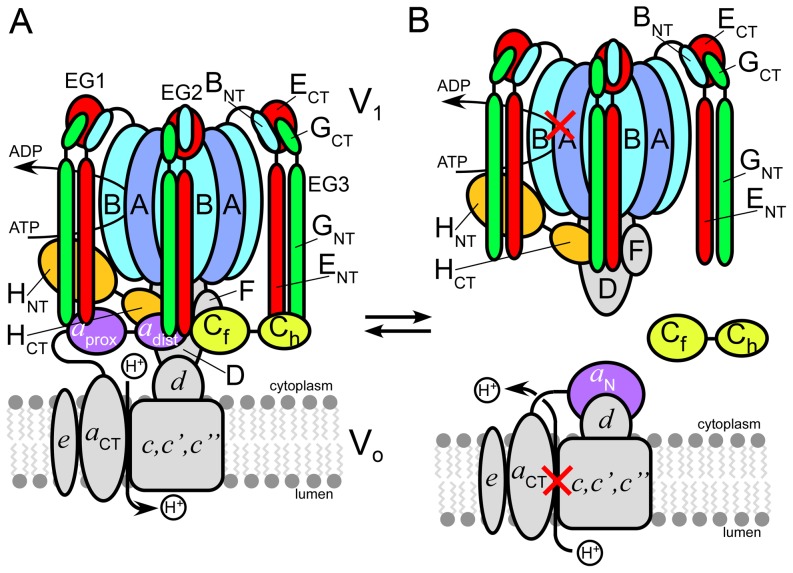
Model of the subunit architecture of eukaryotic vacuolar ATPase. (A) V_1_-ATPase (top) and membrane bound V_o_ proton channel (bottom) are linked by three peripheral stalks (EG1, EG2, EG3) that connect the top of the V_1_ to subunits C, H, and the N-terminal domain of the membrane integral subunit *a* (*a*_NT_). Subunits C, H and *a*_NT_ are two domain proteins with C_head_ and C_foot_, H_NT_ and H_CT_, *a*_NT_(proximal) and *a*_NT_(distal) domains, respectively. (B) During enzyme regulation by reversible dissociation, V_1_-ATPase is released from the membrane bound V_o_ and the activity of both resulting complexes is silenced. To enable enzyme disassembly, protein-protein interactions involving subunits EG, C, H, DF, *d* and *a*_NT_ have to be broken.

V-ATPase is composed of two functional parts, a cytoplasmic ATPase domain called V_1_ and a membrane bound proton channel domain referred to as V_o_. In yeast, the V_1_-domain contains subunits ABCDEFGH with a stoichiometry of 3∶3∶1∶1∶3∶1∶3∶1 [19] and the V_o_ sector is made of subunits *acc'c''de* in the presumed ratio of 1∶8∶1∶1∶1∶1 (**Fig. 1A**). The subunit composition and overall architecture of the V-ATPase is highly conserved from yeast to mammals (except subunit *c'*, for which no mammalian homolog has been found so far), making yeast the model system of choice for studying the enzyme's structure and mechanism.

V-ATPase is a rotary motor ATPase and as in the related F- and A-ATP synthases found in mitochondria, bacteria, chloroplasts and archaea, energy coupling involves rotation of a central stalk made of V_1_ subunits D and F together with the proteolipid subunits *c*, *c'* and *c''* of the V_o_ [20], [21]. However, unlike F- and A-ATPase, eukaryotic V-ATPase is regulated by a reversible dissociation mechanism in which V_1_ disengages from the V_o_ and the activity of both V_1_ (MgATPase) and V_o_ (transmembrane proton conductance) is silenced (**Fig. 1B**). Early studies in yeast [22] and insect [23] indicated that nutrient (glucose) availability is the main trigger for V-ATPase regulation but more recent studies suggest that the signals that lead to disassembly or assembly are more complex [24]–[26]. In higher eukaryotes, factors associated with cell development or tissue maturation as well as interaction with kinases and other enzymes such as aldolase have been implicated in the assembly state of the complex [13], [27]–[29].

Besides the central rotor, intact V-ATPase is stabilized by a ‘stator’ domain composed of peripheral stalks (subunit EG heterodimers) that bind subunits C and H and connect these to the membrane via interaction with the large N-terminal cytoplasmic domain of the V_o_ *a* subunit (*a*_NT_) [30]. Eukaryotic V-ATPase has three peripheral stalks referred to as EG1, EG2 and EG3. Two of the peripheral stalks connect the top of the V_1_ to the membrane integral *a* subunit (EG1 and EG2), with a third one (EG3) connected to subunit C (see **Fig. 1A**). As a result of activity regulation by enzyme disassembly, subunit C is released from both V_1_ and V_o_ and while enzyme disassembly appears to be a spontaneous process, there is evidence that reassembly of the complex, during which subunit C is reincorporated, requires presence of a chaperone called RAVE [31], [32]. A major limiting factor in our understanding of the molecular mechanism of reversible disassembly is the lack of atomic resolution structural information for the eukaryotic V-ATPase complex. While crystal structures for subunits H [33] and C [34] of yeast V-ATPase have been solved, there is currently no high resolution structural information available as to the interactions of these and other subunits in the V_1_-V_o_ interface. Knowledge of these interactions, however, is essential for both a more detailed understanding of the process of reversible enzyme dissociation and for the design of peptides or small molecules that could be used to modulate aberrant V-ATPase activity in the disease state by interference with the assembly or disassembly process.

Previously, we have identified subunit-subunit interactions in the related F- and A-ATPase that were based on in vitro interaction studies between a stator subunit and a short peptide of another subunit of the complex [35]–[37] or between full length subunits or subunit domains of the yeast V-ATPase [38], [39]. Here we have developed a high throughput approach for identifying subunit-subunit interactions in the yeast V-ATPase complex using peptide arrays. V-ATPase subunits were divided into 20 amino acid peptides, which were probed by expressed subunits and domains. This approach has allowed us to identify novel details of the subunit-subunit interactions in the vacuolar ATPase V_1_-V_o_ interface.

## Results

**Figure 2:**
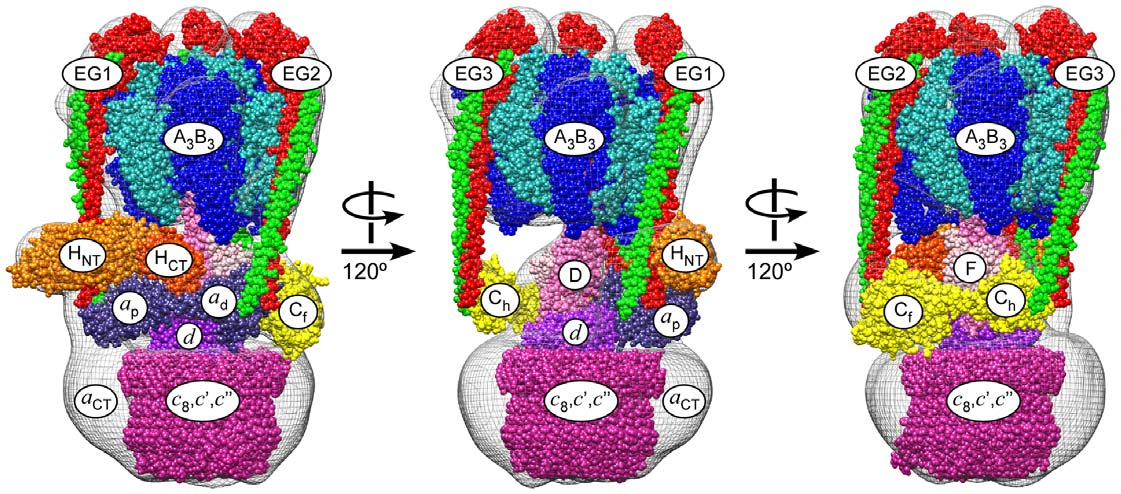
Pseudo atomic model of yeast V-ATPase. Three views of the V-ATPase complex are shown rotated by 120° each. Subunit names are indicated. The model was obtained by fitting available crystal structures into the 3-D EM reconstruction of the yeast V-ATPase complex [30]. The crystal structures used were: A_3_B_3_DF from *T. thermophilus* (3a5c, 3aon, blue/cyan); EG from *T. thermophilus* (3k5b, red/green); Subunit *a* N-terminal domain (*a*_NT_), modeled by threading the yeast primary sequence into the crystal structure of I_NT_ from *M. ruber* (3rrk, violet); Subunits H and C from yeast V-ATPase (1ho8, orange/orange-red; 1u7l, yellow); Subunit *d*, modeled by threading the yeast primary sequence into the crystal structure of *T. thermophilus* subunit C (1r5z, purple); K_10_ ring from *E. hirae* (2bl2, pink).

We recently obtained a pseudo atomic resolution model of the yeast V-ATPase that was built by fitting available crystal structures of V-ATPase and related A-ATPase subunits into the density envelope of a 3-D EM reconstruction (**Fig. 2**) [30]. However, as there are only two available crystal structures for individual yeast V-ATPase subunits (C and H) [33], [34], we have little detailed information on how the stator subunits C, E, G and H bind each other and connect the V_1_-ATPase domain to the N-terminal domain of the V_o_ *a* subunit (*a*_NT_). A schematic of these interactions in the ATPase - proton channel interface and which of these interactions have to be broken during reversible enzyme dissociation is illustrated in **Fig. 1**. To characterize the affinities of subunit-subunit interactions in the V_1_-V_o_ interface, we have recently begun expressing individual yeast V-ATPase subunits and subunit domains for in vitro binding experiments. For example, using isothermal titration calorimetry (ITC), we could show that the head domain of subunit C (C_head_) interacts with EG heterodimer with high affinity [38] whereas the interaction between the distal lobe of *a*_NT_ and EG or the foot domain of subunit C (C_foot_) is relatively weak [39]. In earlier studies with the related F- and A-ATPase, we found that several key subunit-subunit interactions are mediated by short stretches of primary sequence from one of the interacting proteins. For example, using NMR spectroscopy we could show that a peptide comprising the C-terminal 21 residues of the A-ATPase H subunit (equivalent to yeast G) binds tightly to the C-terminal domain of subunit E [34], while the peripheral stalks themselves were found to bind to F- or A-ATPase catalytic domains via interaction with the N-terminal ∼20 residues of the α or B subunit, respectively [36], [37]. In the current study, we have designed a high throughput peptide array based approach for obtaining information on the specific sites of interaction between the individual subunits that constitute the interface between ATPase and proton channel domains in the eukaryotic vacuolar ATPase. Knowledge of both the detailed nature and affinities of the protein-protein interactions in the V_1_-V_o_ interface will be essential for an understanding of the mechanism of V-ATPase activity regulation by reversible enzyme dissociation and will aid with identifying small molecules such as short peptides that may function as inhibitors by interfering with enzyme assembly.

### Design of peptide arrays for V-ATPase subunit-subunit interactions

**Figure 3:**
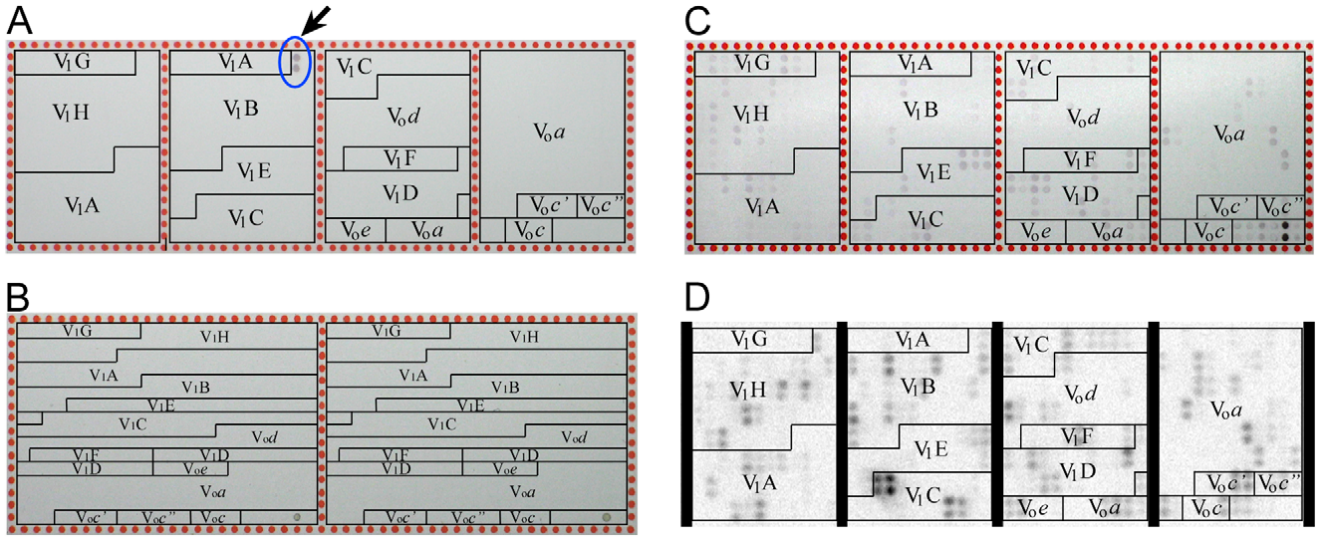
Array design and analysis. (A,B) The two array layouts used for this study. In (A), the duplicate peptide spots were next to each other whereas in (B), spots were duplicated to the same location on the alternate half of the array. The array in (A) was probed with anti subunit B monoclonal antibody (mAb) followed by detection with anti mouse alkaline phosphatase (AP) coupled antibody. As can be seen, two adjacent spots corresponding to the very N-terminal peptide of subunit B were detected (see arrow). The array in (C) was probed with EG-FLAG and binding was analyzed with AP-coupled anti FLAG-mAb. Anti FLAG detection also highlights the control FLAG peptide at the bottom right of the array. The array in (D) was probed with subunit C fused with maltose binding protein (MBP) at its N-terminus. Binding was detected via chemiluminescence from anti MBP mAb coupled to horseradish peroxidase (HRP).

All peptide arrays used in this work were designed in-house by splitting individual subunit primary sequences into overlapping 20-mers. Each peptide was represented by two identical spots on the array, and adjacent spots in the sequence began 10 amino acids apart (except the A&B subunits of V_1_, which were 12 amino acids apart). The arrays, with a total of 768 spots (a duplicate of 378 V-ATPase peptides plus 5 controls and 1 empty spot), were synthesized and spotted onto 25×75 mm glass microscope slides by Intavis, GmbH. Peptides were linked at their C-terminal end to cellulose to give an individual spot density of 5–15 pmol/mm^2^ and with an average spot diameter and thickness of 0.75 and 0.1 mm, respectively, the peptide concentration within the cellulose matrix of the spots was estimated to between 50 and 150 µM. Two array layouts were used, one where the duplicate spots where next to each other and one where the two duplicates were spotted on adjacent areas (**Fig. 3A,B**).

### Antibody probes

To test the applicability of the method, we first probed the arrays using monoclonal antibodies against subunits *a*, A, B, and C, respectively. **Fig. 3A** shows a V-ATPase peptide array probed with anti subunit B monoclonal antibody 13d11. As can be seen, only one doublet of spots corresponding to the N-terminal 20 residues of subunit B was detected by the antibody, suggesting that mAb 13d11 binds to the N-terminus of subunit B (see arrow in **Fig. 3A**). No subunit specific interactions could be detected with mAbs against subunits *a*, A and C, indicating that the epitopes for these antibodies are represented by more complex, 3-dimensional structural elements that are not represented by the one dimensional peptides on the array (not shown).

### V-ATPase subunit peptide array interactions

The majority of V-ATPase subunit probes were expressed in *E. coli* as N-terminal fusions with maltose binding protein (MBP). For the analysis of V-ATPase subunit array interactions, a total of 10 probes were used including subunits (and subunit domains) E, H, H_CT_, C, C_foot_, C_head_ (all as MBP fusions), G (as G-FLAG) and sub complexes EG and V_1_-ATPase (detected by FLAG-G and G-FLAG, respectively). Peptide arrays were exposed to purified subunits or subunit domains at a concentration of 4 µM and the resulting binding to the arrays was probed via the fusion partner (MBP) or a FLAG tag (in case of subunit G and intact V_1_). Incubation of arrays with purified MBP only followed by immuno detection as for subunit fusions served as a reference (negative control) in the analysis of the MBP subunit fusion array interactions. No significant signal was detected with antibody only as probe (not shown). The antibodies used to detect bound subunits were coupled to horseradish peroxidase (HRP) and the resulting chemiluminescence signal was recorded on a fluorescence scanner. We also used alkaline phosphatase (AP) coupled antibodies for detection (**Fig. 3C**) but due to the superior dynamic range of the HRP system, all arrays used in the subsequent analysis were developed using chemiluminescence detection (**Fig. 3D**). Digital images of the arrays were processed and the resulting data sets were imported as spreadsheets for data analysis using the TIGR Multi-Experiment Viewer (MeV) software package [40], [41]. To ensure statistical significance, between two to eight duplicate array experiments were performed for each subunit probe, providing 4–16 independent measurements as each array contained two spots per peptide.

### Developing a V-ATPase interaction ‘heat map’

After importing data into MeV, the most effective way to cluster the data regarding the behavior of similar protein probes is to use the Analysis of Variance (ANOVA) method. ANOVA allows the specification of distinct groups of varying sizes, ultimately followed by clustering on similarity of peptides, probes, or both. The initial ANOVA as determined in the TIGR Multi-Experiment Viewer (MeV) [40], [41] showed trends in the similarities in interaction signal for the arrays of the combined data set. In the case of probes where the interaction data of at least two arrays were in clear agreement on a clustered set, arrays that deviated significantly were not included in the further analysis. Also excluded were arrays that showed overall poor signal to noise or blurring of the chemiluminescence signal. The ANOVA was further supported by hierarchical clustering (HCL) performed on the result; if a two-dimensional HCL run on ANOVA results produced the expected sample clusters after removing a poorly clustering sample, the removed sample was considered to be flawed.

**Figure 4:**
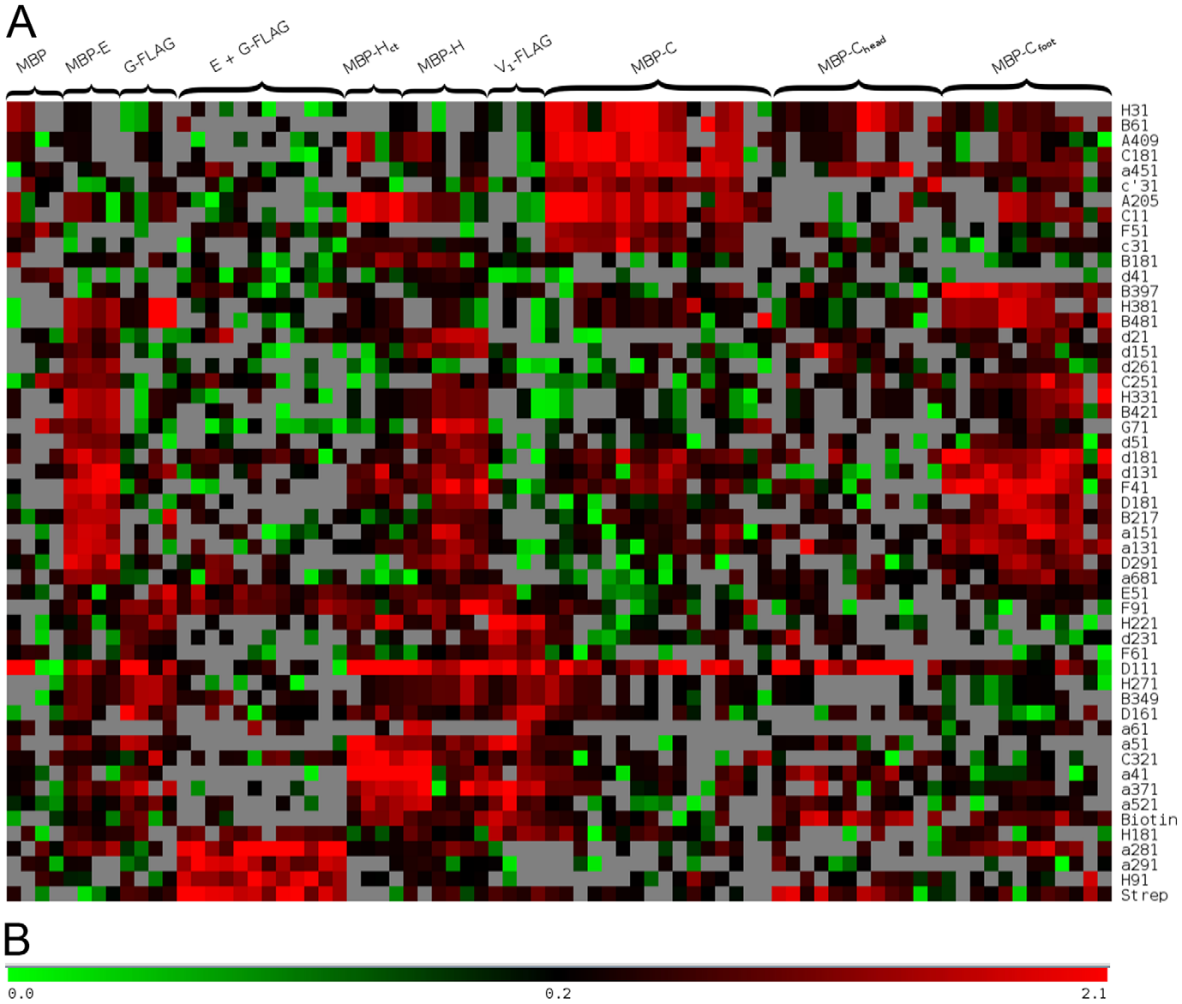
Interaction heatmap of the V-ATPase peptide array analysis. (A) The heatmap was generated from the V-ATPase peptide array data within MeV software using the ANOVA and HCL protocols. The probes are indicated by brackets along the top of the heatmap and the consensus peptides are indicated on the right. (B) Color scale for the interaction score. A cut off of 1 was used to generate the list of peptides shown in (A). For details, see text.

Using the parameters described earlier, we obtained a heat map with 53 peptide spots that were selected as significant (**Fig. 4A**). Included in this collection were the two control peptides, biotin and strep tag and one V-ATPase peptide spot (D111, amino acids 111–130 of subunit D) that was detected by most subunit probes including MBP; interactions from these three spots were automatically excluded from further analysis. This leaves a set of 50 peptides for further evaluation. One further correction that is made with this data is that in order for an interaction to be considered significant for our analysis, peptides were only accepted where at least 75% of the probes listed had an (RMS-divided) interaction score of at least 1 (**Fig. 4B**).

Overall, a total of 9 different protein subunits, subunit domains, or subunit assemblies were used as probes. In the following section, a more detailed description of the individual probes and their corresponding array based ‘intra-actome’ is provided. In the text, peptides are listed by subunit name followed by the first residue number. Also, interactions to peptides that were part of the hydrophobic core of the large A and B subunits were considered non specific and are not discussed further. See Table 1 for a summary of the interaction results.

**Table 1:** Summary of the interactions considered significant by MeV software.

Subunit	Size (KDa)	Length (AA)	Tag	Hits	V_1_ Hits	V_O_ Hits
G	13	114	Flag	7	**H(221-240)**, **H(271-290)**, B(349-368), F(91-110), D(111-130)*, D(161-180)	**a(371-390)**
H	54	478	MBP	12	G(71-90),H(271-290), B(349-368),C(321-340), F(41-60),(91-110),D(111-130)*	d(21-40), d(51-70), d(131-150), **a(41-60)**, **a(51-70)**
H_CT_	14	126	MBP	8	H(221-240), A(205-224), C(11-30), C(321-340), D(111-130)*	**a(41-60)**, **a(51-70)**, a(371-390), a(521-540)
C	42	392	MBP	8	A(205-224), A(409-428), B(61-80), C(11-30), C(181-200), D(111-130)*	a(451-470), c^(^'^)^(31-50)
C_head_	11	98	MBP	1	D(111-130)*	
C_foot_	29	257	MBP	10	H(381-400), B(217-236), B(397-416), F(41-60), D(181-200)	d(131-150), d(181-200), **a(131-150)**, **a(151-170)**, **a(281-300)**
E	26	233	MBP	15	**H(331)**, G(71-90), F(41-60), D(181-200), D(291-310), B(421)	d(21-40), d(51-70), d(131-150), d(151-170), d(181-200), d(261-280), **a(131-150)**, **a(151-170)**, **a(281-300)**, a(681-700)
EG	39	-	Flag	6	**H(91-110)**, **H(181-200)**, E(51-70), F(91-110)	**a(281-300)**, **(291-310)**
V_1_	∼600	-	Flag	8	H(221-240), F(61-80), D(111-130)*, D(161-180)	d(231-250), **a(41-60)**, **a(51-70)**, **(371-390)**

**Figure 5:**
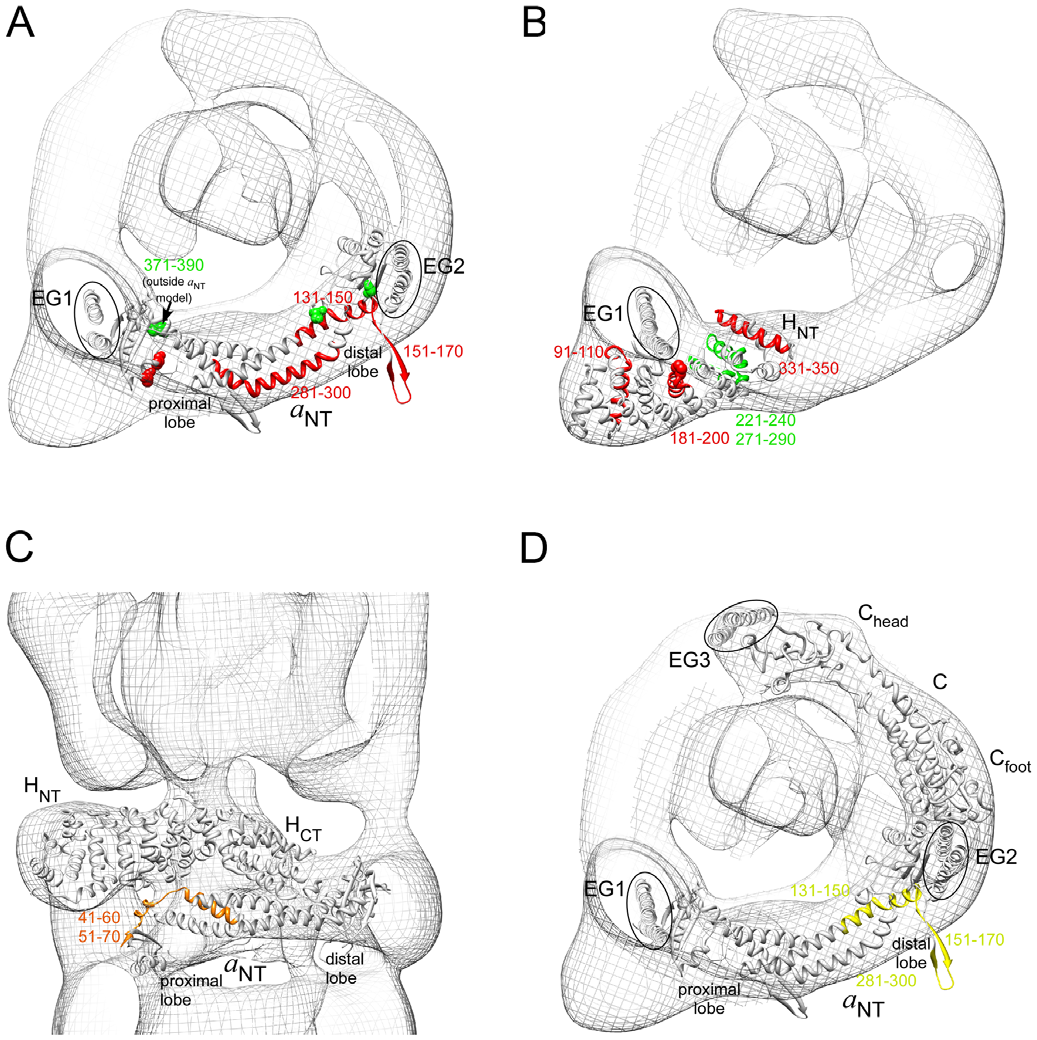
Modeled subunit interactions based on the array analysis. Predicted interactions were highlighted for subunits for which crystal structures or homology models are available. Interactions between EG and *a*_NT_ (A), EG and H_NT_ (B), H and *a*_NT_ (C) and C and *a*_NT_ (D). The residues highlighted by spacefill indicate sites from photo crosslinking to adjacent subunits was observed in studies from the Forgac laboratory [49], [58], [60]. For details, see text.

Interactions in bold are illustrated in **Fig. 5**. The interaction denoted with an asterisk are to the promiscuous D111 peptide.

### Interactions of V-ATPase peripheral stalk subunits

The V-ATPase has three peripheral stators consisting of the E and G subunits bound in a 1∶1 configuration [19], [30]. While the current model shows the approximate locations of these stators within the intact V-ATPase complex (**Fig. 2**), the molecular details of their interactions are not well understood. To examine these interactions, three constructs were used – subunit E with an N-terminal MBP, subunit G with a C-terminal FLAG, and a heterodimer of an untagged subunit E with subunit G with a N-terminal FLAG.

Subunit G (Vma10p) is a 13 kDa, 114 amino acid protein that is modeled primarily as an extended α helical structure (a crystal structure for the bacterial A/V-ATPase homolog of subunit G is available [42]). Earlier studies in yeast showed that subunits E and G interact strongly [43], [44] and that archaeal subunit G binds the N-terminal 100 residues of subunit E in an α helical coiled coil followed by a globular C-terminus that also binds the C-terminal 20 residues of G [42], [45]. Despite its established ability to bind subunit E, we did not observe significant binding of subunit G to any of the peptides of the E subunit on the array. This unexpected finding may be explained by the earlier observations that purified subunit G has a tendency to aggregate [19], [38], that subunits E and G do not bind each other in vitro, and that in order for heterodimer formation to occur, both subunits have to be co-expressed in e.g. *E. coli* [38], [46].

Of the other subunits of the V-ATPase subunit G showed an interaction with were subunit H(221, 271), B(349), F(91), D(161) and *a*(371-390). The interactions with subunit H are discussed in more detail below. For the interactions with D and F, see above and Discussion.

Subunit E (Vma4p) is a 26 kDa, 233 amino acid two domain protein that binds subunit G via its α helical N-terminus (see above). As an individual protein with an N-terminal MBP fusion, subunit E showed a total of 15 interactions with other V-ATPase subunits, including one interaction with subunit G(71) that can be rationalized based on the known EG heterodimer formation [38]. Of the other 14 interactions, 9 were to subunits of the rotor domain including D(181, 291), F(41) and *d*(21, 51, 131, 151, 181, 261), one to H(331) and one to B(421). The 9 interactions mentioned first are counterintuitive as they would suggest a linkage of peripheral stator and central rotor; hence the possibility that at least some of these interactions are non specific in nature cannot be excluded at this point. There are also three interactions for subunit E to subunit *a* which are not seen when probing with subunit G; all three of these – *a*(131, 151, 281) – are in the soluble N-terminal domain of subunit *a*, and furthermore, are within or next to the proposed coiled-coil domain of the same (see below).

The EG heterodimer was prepared by co-expression of both subunits in *E. coli*, with MBP on the N- and a FLAG tag on the N-terminus of subunit G. We have recently shown that EG heterodimer binds to the head domain of subunit C (C_head_) with high affinity [38], however, since there are three copies of EG in the V-ATPase, binding sites to other subunits including H, B and the N-terminal domain of *a* (*a*_NT_) are predicted to exist (**Fig. 2**). When used as a probe on the arrays, the heterodimer showed a reproducible interaction with 7 peptides, including the strep control peptide (adjacent to the FLAG control peptide) and peptide F(91-110) that was also seen with individually expressed G subunit as probe. Of the remaining 5 peptides, one was to subunit E(51), and two each were to subunit H(91, 181) and the N-terminal domain of subunit *a*(281, 291).

The interactions observed between subunits E and G and the N-terminal soluble domain of subunit *a* and subunit H are summarized in **Fig. 5A,B**. For interpreting the EG interactions to subunit *a*_NT_, the yeast primary sequence was threaded into the recent crystal structure of the N-terminal domain of a bacterial homolog to V-ATPase subunit *a* (I_NT_, [47]). **Fig. 5A** shows a cross section of the EM model as seen from the cytoplasm towards the membrane with the *a*_NT_ model and the N-terminal portions of the two EG heterodimers (EG1 and EG2 highlighted by ovals). Subunit *a*_NT_ is a two domain protein with proximal (amino acids 14–53, 331–406) and distal lobes (183–256) connected by an α helical coiled coil (107–152, 267–330). Based on EM reconstructions [30], [48], EG1 is predicted to bind at the proximal and EG2 at the distal lobe of the subunit (see Fig. 1A and Fig. 2, left panel). As can be seen, both the proximal and distal lobes of *a*_NT_ have peptides that showed an interaction with either G (in green) or E/EG (in red), sites that are near amino acid residues that were previously shown to be in close proximity to E and G subunits based on photo chemical crosslinking studies [49] (crosslinks to E and G indicated by red and green space fill residues in **Fig. 5A**, respectively). **Fig. 5B** shows a cross section of the EM model highlighting peptides in the N-terminal domain of subunit H (H_NT_) that showed an interaction with E (in red) or G (in green). As can be seen, the position of the peptides is consistent with the EM fit and earlier crosslinking studies that put Pro197 in close proximity to subunit E [**49**].

Subunit H (Vma13p) is a 54 kDa, 478 amino acid subunit required for activity but not assembly of the intact V-ATPase [50]. Subunit H is an α helical two domain protein with an N-terminal importin like structure (1–352) and a C-terminal globular domain (353–478) connected by a linker peptide [33]. N-terminal MBP fusions of full-length subunit H and its C-terminal domain (H_CT_) were used to probe the arrays as earlier work showed that H_CT_ functions independently of H_NT_ in enzyme regulation [51]. Full-length H shows 12 significant interactions, including one each to subunits B(349), C(321) D(111) and G(71), two to F(41, 91), four to *d*(21, 51, 131, 151) and two to the N-terminal domain of *a*(41, 51). Using H_CT_ as a probe lead to a set of 8 interacting peptides. Included in this set were four spots that were also seen when full-length H was used as a probe – C(321), D(111) and *a*(41, 51). Interactions to H_CT_ that were not seen with full-length H were A(205), C(11), H(221) and *a*(371). **Fig. 5C** shows modeling of the peptides in the proximal lobe of *a*_NT_ that showed an interaction to both full length H and H_CT_. As can be seen, the sequential peptides *a*(41-60; 51-70) are located in the H-*a*_NT_ interface where they would provide a large portion of the interacting surface between the two subunits. For the subunit H interactions with subunits for which there is no crystal structure as well as interactions between subunits H and C, see below.

Subunit C of the V-ATPase is a 42 kDa, 392 aa protein with two distinct domains, C_foot_ (amino acids 1–151; 287–392) and C_head_ (166–263) [34]. Earlier work has shown that individually expressed C_foot_ and C_head_ domains are stably folded [38]. Full-length C subunit with an N-terminal MBP fusion showed a total of 8 interactions, with two to subunit A(205, 409), one to B(61), two to subunit C itself (11, 181), one to D(111), one to subunit *a*(451), and one to a cytoplasmic loop of the proteolipid subunit *c*(31).

While purified C_head_ yielded only the D(111) peptide as consistently interacting, probing with isolated C_foot_ gave a set of 10 interacting peptides, with 5 to V_o_ subunits *a*(131, 151, 281) and *d*(131, 181) and the remaining 5 to V_1_ subunits B(217, 397), D(181), F(41), and H(381). **Fig. 5D** shows modeling of the interactions between subunit C and the N-terminal domain of subunit *a*. The sequence *a*(131-170) includes the end of the N-terminal half of the coiled coil and most of a loop sequence in the distal lobe that is not present in bacterial I_NT_.

In addition to individual subunits and subunit domains, the V-ATPase peptide arrays were also probed with intact V_1_-ATPase that was affinity purified from yeast via a FLAG tag fused to the N-terminus of subunit G. The V_1_-FLAG probe showed interactions with 8 peptides, including D(111, 161), F(61), H(221), *d*(231) and *a*(41, 51, 371). These interactions closely resemble the ones observed with isolated G and H subunits. It should be noted here that V_1_ purified from yeast does not have C subunit bound [19].

## Discussion

The proton pumping activity of the eukaryotic vacuolar ATPase is regulated by reversible enzyme dissociation, a unique mechanism during which the soluble V_1_-ATPase disengages from the membrane bound V_o_-proton channel domain and the activity of both domains is silenced [22], [23], [51], [52] (see **Fig. 1B**). A single copy subunit, C, is released from both V_1_ and V_o_ during disassembly and recruited back upon receipt of the signal for reassembly [53]. Key to understanding the mechanism of V-ATPase reversible dissociation will be a detailed knowledge of the protein-protein interactions in the interface between V_1_-ATPase and ion channel. We have recently obtained a pseudo atomic model of the yeast V-ATPase by fitting available crystal structures of yeast V-ATPase subunits C and H as well as structures from related rotary motor ATPases into the EM density map of the complex [30]. The resulting model predicted that functional coupling between V_1_-ATPase and V_o_ proton channel domain in intact V-ATPase is mediated by interactions between components of the rotating central stalk (subunits D, F and subunit *d*) as well as interactions involving three EG heterodimer peripheral stalks (EG1, EG2, EG3) that connect the catalytic A and B subunits of the V_1_ to the C and H subunits and the cytoplasmic N-terminal domain of the membrane bound *a* subunit (**Fig. 2**). However, due to the limited resolution of the EM model and the fact that crystal structures for only two V-ATPase subunits are available, the precise nature of these interactions are not known.

To obtain more detailed information on the coupling of V_1_ and V_o_, we have employed a peptide array based approach to identify interactions between subunits in the eukaryotic vacuolar ATPase from the yeast *S. cerevisiae*. Protein-protein interactions have been investigated in the past using peptide arrays, and this technique has been shown to be useful in finding interaction sites using intact proteins as probes [54]–[56]. The linear nature of a peptide excludes the identification of certain interaction types (such as three dimensional epitopes, or binding sites involving post-translational modifications), however, as mentioned above, we previously observed that some key subunit-subunit interactions in the related F- and A-type rotary motor ATPases are driven by short peptides [35]–[37]. To investigate the possibility that similar interactions exist in the yeast V-ATPase, we designed a high throughput approach using peptide arrays, focusing on interactions involving stator subunits C, E, G, H and the N-terminal domain of subunit *a* (*a*_NT_). Most of these subunits were previously found to be folded as two domain proteins: C_foot_ and C_head_ for subunit C [34], H_NT_ and H_CT_ for H [32], EG_NT_ and EG_CT_ for EG heterodimer [35], [42] and *a*_proximal_ and *a*_distal_ for the N-terminal domain of subunit *a* [47]. For probing the arrays, we used both full length subunits as well as their domains, where available.

On the V-ATPase peptide arrays, each subunit is represented by a duplicate of 20 amino acid peptides with a 10 amino acid overlap (a smaller overlap was used for the large catalytic subunits A and B), resulting in a ∼2X coverage of the entire primary sequence of the close to 1 MDa complex. Interactions to native subunits were then probed by exposing the arrays with *E. coli* expressed yeast V-ATPase subunits (or intact V_1_ purified from yeast). Interactions were detected either via maltose binding protein fused to the N-terminus of subunits or, in case of subunit G, via a FLAG tag fused to its N- or C-terminus. N-terminal FLAG or MBP fusions were used for the interaction studies as there are no commercial antibodies available for most of the subunits used in this study and because we previously found that enzyme assembly and subunit-subunit interactions are not affected to a significant degree by N-terminal fusions.

The torque of rotational catalysis in yeast V-ATPase is ∼40 pN•nm (generated by the free energy change of ATP hydrolysis of ∼40 kJ/mol [57]), a force the interactions between V_1_ and V_o_ have to be strong enough to counteract. At the same time, in eukaryotic V-ATPase, at least some of the interactions have to be weak enough to allow rapid breaking during activity regulation by reversible enzyme disassembly [39]. Considering these two seemingly contradictory requirements, we therefore expected that some of the interactions involving the structural linkage between V_1_ and V_o_ to be weak and some to be strong, in line with our recent work where we found a high affinity interaction between EG and C_head_ [38] and low affinity interactions between *a*_NT_ and both C_foot_ and EG heterodimer [39]. From the estimated amount of peptide contained in each spot, interactions with K_d_s in the nanomolar to tens of µM range should be detectable on the arrays used in our study.

Initial attempts to analyze individual arrays manually were hampered by variations in chemiluminescence staining intensities and scanning resolution. However, implementing statistical analysis with HCL and ANOVA methods [40], [41] allowed us to rapidly search for patterns in data that were not otherwise immediately apparent. The same method was also a valuable tool for post-analysis array qualification; arrays which gave signal patterns that were distinctly different from others of the same probe were easily identified and excluded from the analysis. The array layout where each peptide spot was represented twice provided some initial assessment of the reproducibility of the signal, however it was found that rather than using the duplicates to verify signal on the array, it was more useful to handle each array as parallel runs and hence import the arrays as two independent experiments. The advantage of this was that then if one duplicate spot was dramatically lower – or completely absent – in signal in comparison to its twin, that would not throw off the latter interpretation of the signal set.

The final assessment of the combined array interaction data analysis comes from mapping interactions onto the current structural model of yeast V-ATPase (**Fig. 2**) for subunits where crystal structures (C, H) or homology models (*a*_NT_) are available. Starting with interactions involving the C subunit it is noted that C_head_ showed no significant specific interactions with peptides of other V-ATPase subunits except a peptide corresponding to residues 111–130 of the D subunit (‘D111’). As can be seen from the heatmap shown in **Fig. 4A**, D111 showed promiscuous interaction with essentially all the probes used, suggesting that binding to this especially ‘sticky’ peptide may not be physiologically relevant. We recently showed that C_head_ forms a high affinity ternary complex with EG heterodimer in vitro [38]. The lack of any specific interaction of C_head_ with any peptides on the array (except D111) is likely because the interaction of C_head_ with EG depends on amino acid residues from both E and G subunits. C_foot_ on the other hand was shown to be involved in a low affinity interaction with the proximal lobe of the N-terminal domain of subunit *a*, for which a K_d_ of 30 µM was measured [39]. Array analysis with MBP-C_foot_ showed a reproducible interaction with three peptides of subunit *a*, all of which are located in or near the subunit proximal domain of the subunit. Of special interest is the sequence 131–170 (covering two peptides) that contains a loop sequence not present in the homologous subunit of the bacterial A/V-type ATPase, for which a crystal structure is available [47]. As the bacterial enzyme has no subunit C, it seems plausible that the corresponding loop in *a*_NT_ represents the binding site for C_foot_, consistent with photochemical cross linking [58] and placement of the subunits based on the EM model [30] (**Fig. 5D**). Interestingly, the same peptides showed an interaction with subunit E, consistent with our earlier finding that the proximal lobe of *a*_NT_ interacts with both C_foot_ and EG heterodimer in a ternary complex [39].

The EM model shown in **Fig. 2** predicts that the N-terminal domain of subunit H is in contact with peripheral stalk EG1, a prediction that is also supported by earlier two hybrid and in vitro interaction studies using recombinant proteins [51], [59]. Consistent with that prediction, subunits G, E and EG heterodimer showed an interaction with subunit H N-terminal peptides near Pro197, a residue that had previously been shown to be in close contact with EG heterodimer [60]. In addition, peptide 271–290 contains several residues that are highly conserved from yeast to human (283-KEKVxR-288) [33], further support for the involvement of this region of H in EG binding (**Fig. 5B**).

Consistent with earlier reports of in vitro binding of the C-terminal domain of subunit H and the N-terminal domain of subunit *a* [61], an interaction was observed between both full length subunit H and its C-terminal domain, H_CT_, and two overlapping peptides of the N-terminal domain of subunit *a* covering residues *a*41-70. In the structural model of yeast subunit *a* N-terminal domain, the peptides are positioned where they are predicted to be in contact with both domains of the H subunit (**Fig. 5C**). Subunit H has a dual function in the V-ATPase: in the intact enzyme, subunit H is required for ATP hydrolysis driven proton pumping but not for assembly [50] while in dissociated V_1_, the subunit, via it's C-terminal domain, functions to silence MgATPase activity [51], [52]. Both intact subunit H and H_CT_ showed an interaction to subunit F peptides on the array, interactions that can be rationalized with the earlier proposal based on cross linking that silencing of MgATPase activity in V_1_ is caused by an interaction of stator and rotor subunits H and F, respectively [60].

EG heterodimer and its component subunits E and G show interactions to subunit H (see above) as well as the N-terminal domain of subunit *a* and subunit C. Based on the EM model, both EG1 and EG2 heterodimers are predicted to bind *a*_NT_, EG1 at its proximal and EG2 at its distal lobe. Subunit *a*_NT_ peptides that showed an interaction with E, G and EG include residues in both distal and proximal lobes (**Fig. 5A**), close to where EG1 and 2 are predicted to bind, and close to sites that have previously been cross-linked to E and G [49]. However, an interaction was also seen to peptides from the coiled coil domain that connects the two lobes of *a*_NT_ and it cannot be ruled out that this interaction, detected mainly with isolated E and C subunit, is due to nonspecific pairing of amphipathic α helices (see below).

Aside from individual subunits, the EG subcomplex and subunit domains, we also probed arrays with intact V_1_-ATPase purified from yeast via a FLAG tag fused to the N-terminus of the G subunit. We previously showed that V_1_ isolated this way contains subunits A, B, D, E, F, G, H in a ratio of 3∶3∶1∶3∶1∶3∶1 [19]. Not surprisingly, the interactions detected for the V_1_ probe closely resemble those for isolated G and H subunits. Especially the interaction between V_1_ and *a*_NT_ peptides is interesting as these interactions suggest that binding of cytoplasmic V_1_ to V_o_ in the vacuole may be initiated by binding of subunit H to *a*_NT_ as illustrated in **Fig. 5C**.

As a target, V_o_ subunit *a* (as expressed by gene Vph1) revealed several notable interactions, mainly from peptides of its soluble N-terminal domain to peripheral stalk subunits E, G, H and C (see above). Beyond these interactions with peptides of the soluble domain, there were three interactions to the C-terminal, membrane bound domain. A recent model of the C-terminal domain of yeast subunit *a* shows 8 transmembrane α helices as well as several notable loops on both the lumenal and cytoplasmic sides [62]. Full-length C subunit showed an interaction with *a*451-470 (connecting TM2 and TM3), and subunit E bound to *a*681-700 (connecting TM6 and TM7). These two peptides are predicted to be on the cytoplasmic side and it is possible that the corresponding interactions to E and C help stabilize the stator domain. A third peptide (*a*521), predicted to connect TM3 and TM4 showed an interaction to H_CT_ and V_1_ but since this peptide is predicted to be on the lumenal side of the membrane, it is possible that this interaction is a false positive.

In summary, the peptide array data presented here revealed detailed information for a number of potential subunit-subunit interactions that are consistent with current models of V-ATPase architecture including interactions between stator subunits EG, C, H and the N-terminal domain of subunit *a*. However, along with these key interactions (involving stator subunits at the V_1_-V_o_ interface) that can be rationalized with information from the structural model and other biochemical data, the array analysis presented here also revealed a number of interactions that are not easily explained based on current knowledge about V-ATPase structure. Some of these interactions may be nonspecific, involving the highly α helical subunits such as H or EG that may bind peptides from other subunits with high α helical content such as D, *d* or *a*_NT_. Other interactions between subunits that are not in direct contact in the EM model may represent possibly transient assembly intermediates such as those described for a complex between subunits H and C based on a recent cryo EM analysis of yeast V_1_-ATPase [63], an interaction also supported by SPR analysis of recombinantly expressed subunits of the human V-ATPase [64].

The array study presented here has revealed a number of peptides that may be involved in key interactions within the V_1_-ATPase - V_o_-proton channel interface, especially within subunits H and *a*_NT_. Ultimately, the physiological significance of the interactions that can be interpreted in context of the present V-ATPase structural model will have to be verified by a more thorough biophysical characterization as well as phenotypic analysis of mutations designed to either weaken or strengthen binding of the involved subunits. These studies are ongoing in our laboratory.

## Materials and Methods

### Reagents

All reagents were from Sigma, unless otherwise noted. Peptide arrays were ordered from Intavis AG, Köln, Germany. Anti-MBP (Maltose Binding Protein) antibody was from New England Biolabs and Enhanced Chemiluminescence (ECL) kit from Perkin-Elmer. Anti-mouse-AP conjugate antibody was obtained from Bio-Rad. Protein subunit H constructs fused with MBP were provided by the laboratory of Dr. Patricia Kane, SUNY Upstate Medical University.

### Plasmid Construction

Yeast V-ATPase Vma4 (subunit E) and Vma10 (subunit G) were cloned into pMAL c2e with MBP fused to the N-terminus of subunit G followed by a ribosome binding sequence and tagless subunit E. Expression of the resulting plasmid was driven by a single promoter. For detection of EG heterodimer, a FLAG tag (DYKDDDDK) was fused to the N-terminus of subunit G. The resulting bicistronic construct was subsequently used to obtain both purified EG heterodimer as well as free FLAG-G. Integrity of plasmids was confirmed by DNA sequencing in the Upstate Medical University DNA sequencing core facility.

### Protein Expression and Purification

MBP-fused individual subunits and subunit pieces were purified using the method provided by New England Biolabs. After amylose column chromatography, the MBP was left attached to the protein of interest, to be used later for detection. Low molecular weight contaminations (from incomplete translation or post lysis degradation) were removed by ultrafiltration (50 kDa cut-off) or size exclusion chromatography as described [38], [39].

Purification of the EG heterodimer was done with three chromatographic steps. First, the MBP fusion was isolated using amylose affinity chromatography according to the manufacturer's instructions followed by PreScission protease cleavage of MBP from the N-terminus of subunit G. The cleaved fusion was dialyzed overnight against 50 mM tris-HCl, 1 mM EDTA, pH 7.9 followed by DEAE sepharose anion chromatography using an AKTA FPLC system (GE Healthcare). MBP, EG and G-FLAG were eluted with a linear (0–500 mM) gradient of sodium chloride. EG and G-FLAG containing fractions were pooled and free MBP was removed by passing the solutions through a 5 ml amylose column. Pure MBP eluted from the column with 10 mM maltose served as control probe for the array analysis.

FLAG-tagged V_1_ complex was purified as described [65] and MBP-E was obtained according as in [19]. Subunit C, C_foot_ and C_head_ MBP fusions were purified as in [38]. SDS-PAGE of the subunits and subunit domains used in this study is shown in **Fig. S1**.

### Array Design

All V-ATPase-derived spots on the peptide arrays are amino acid 20-mers, tethered to the array by a cellulose linker at their C-terminus. Long peptides were chosen over shorter sequences in the interest of improving the chance of the peptides presenting simple structural elements (short α helices and turns in particular) to the probes and enhancing the chance of an interaction. The cellulose linker is employed by the manufacturer with the intent of making more of the peptide accessible than if they were spotted directly on the glass surface. The arrays are affixed to 25 mm×75 mm microscope slides (**Fig. 3**).

For all subunits, each peptide starts 10 amino acids after its predecessor (except for the large subunits A and B where it was 12); hence the first peptide of a subunit starts with residue 1 and the second with residue 11, resulting in an average two-fold coverage of the primary sequence.

The arrays also included Biotin, Strep-tag, HA-tag, c-myc, and FLAG tags as controls, as well as an empty (no peptide) spot. Every spot was present twice on the array and depending on the layout of the arrays the two spots were either next to itself, or transposed to the opposite half of the array.

### Array Experiments

The free MBP obtained as a byproduct of EG heterodimer and G-FLAG purification served as control probe on the arrays and for determining the detection range using anti MBP monoclonal antibodies. MBP was spotted by hand first onto nitrocellulose, to be blocked in 2% nonfat milk in TBS-T and subsequently resolved on a Typhoon 9410 multimode imager (GE Biosciences) in chemiluminescence mode using the ECL kit from Perkin-Elmer. Once the appropriate concentration of MBP was found for array detection, later MBP-based experiments were done with the same molar protein concentration. While some of the array analysis was done using alkaline-phosphatase (AP) conjugated antibodies with BCIP (colorimetric) detection (see **Fig. 3C**), it was found that the signal range of the HRP-based chemiluminescence detection was more linear and reproducible.

Array experiments were done at 4°C on a platform shaker up to the ECL step, using a process similar to Western Blotting. First, the array is blocked in 2% nonfat milk for 1 hour. Then, three 5 minute washes were done in tris buffered saline (TBS), followed by two 5 minute washes in TBS+0.1% Tween-20 (TBS-T). The probe protein was then applied for 1 hour in TBS. Then the same wash sequence was done as before. This was followed by 1 hour of Anti-MBP-HRP, diluted 1∶5,000 in TBS-T. The same wash sequence was then done again after the antibody. After the final wash, binding of anti MBP antibody was detected using chemiluminescence.

### Array data analysis

The TIF images from the Typhoon scanner are first opened using the GIMP software, to find the defined regions of the array on the image. First generation arrays are divided into quadrants, while second generation are in halves. GIMP is used to find the spots, define the regions of the array, align the array properly for a grid, and finally to discard the portion of the image that is not within the spotted area. Following the image analysis in GIMP, the modified image were opened in Image Quant Total Lab (IQTL – GE Biosciences). In IQTL the spots are found on a grid, and quantified. The numbers from IQTL are then imported into a spreadsheet for further analysis.

Data from each array were imported into an individual spreadsheet first, where the data is sorted out into a linear fashion and duplicate spots can be analyzed against each other. Data points from the individual spreadsheets were then transferred to a larger master spreadsheet where each array is listed twice, once for each duplicate of the spot. The master spreadsheet is finally exported as a text file for statistical analysis of the array data using the TIGR MeV4 software.

All statistical interpretation was done using Multi Experiment Viewer (MeV) from The Institute for Genomic Research (TIGR) [40], [41]. First, the text spreadsheet is opened in MeV and Hierarchical Clustering (HCL) is applied to find the relationships between samples of the same probe. Except the MBP-only runs, samples that are shown to cluster poorly or not at all with other runs of the same probe are considered for rejection from the set for further analysis. As the TIGR MeV4 software is designed with DNA microarrays in mind, the spots from our peptide arrays are referred to as “genes” in the program.

After HCL, the next step is to normalize the data so that the ranges for each sample are comparable. This is done through the “Divide columns by RMS” option. Once this is complete, the color scale for the data is readjusted to meet the new range. HCL is run again after this to see the effect this has on same-sample behavior.

Once the data normalization and initial HCL is complete, Analysis of Variance (ANOVA) was employed to see how known sets of runs behave. Our complete data set has 10 groups for ANOVA, each group having at least two arrays (4 columns from the spreadsheet). The groups consist of MBP only, E, G, EG, H, H_CT_, V_1_, as well as C, C_foot_, and C_head_. ANOVA is run with a Standard Bonferroni Correction and a p-value cutoff of 0.05. HCL is then performed on both the significant and non-significant clusters of peptides that are identified by this procedure. The p-value of 0.05 for ANOVA was chosen by comparing the lists of peptides selected as significant for a number of different p-values, and comparing those that were included versus a p-value of 0.01. For p-values of 0.01, 0.05, 0.1, 0.2, and 1.0, the total number of significant peptides selected by ANOVA – including control spots and D111 – were 44, 53, 61, 65, 71, and 202, respectively. Amongst these sets, the peptides selected by a p-value of 0.05 had the lowest number of significant peptides that were excluded by our earlier stated criteria of 75% of same-probe spots scoring at least 1.0, without excluding qualifying probes found for higher p-values.

### Molecular Modeling

All molecular modeling presented in this paper was done in Chimera [66]. A model for the V-ATPase was constructed based on fitting available crystal structures (when available) or homology models of V-ATPase subunits into the 3-D EM map for yeast V-ATPase [30]. The structures shown in this paper are 1u7l (yeast subunit C) [34], 1ho8 (yeast subunit H) [33], N-terminal domain of subunit *a* threaded into the homologous bacterial protein form M. ruber (3rrk) [47], *T. thermophilus* equivalents to E and G (3k5b) [42], A_3_B_3_DF from *T. thermophilus* (3a5c, 3aon) [67], [68], subunit *d* - modeled by threading the yeast primary sequence into the crystal structure of *T. thermophilus* subunit C (1r5z) [69], and the K_10_ ring from *E. hirae* (2bl2) [70].

## Supporting Information

Figure S1 **SDS-PAGE of subunit probes used in this study.** Left lane, molecular mass marker; (1), MBP; (2) MBP-E; (3) FLAG-G; (4) EG-FLAG; (5) MBP-H_CT_; 6 MBP-H; (7) MBP-C; (8) MBP-C_head_; (9) MBP-C_foot_.
(TIF)
