# Supplementary figures and images for: Probing Subunit-Subunit Interactions in the Yeast Vacuolar ATPase by Peptide Arrays
Source: PLoS One. 2012 Oct 12;7(10):e46960. doi: 10.1371/journal.pone.0046960 (PMC3470569; doi:10.1371/journal.pone.0046960)

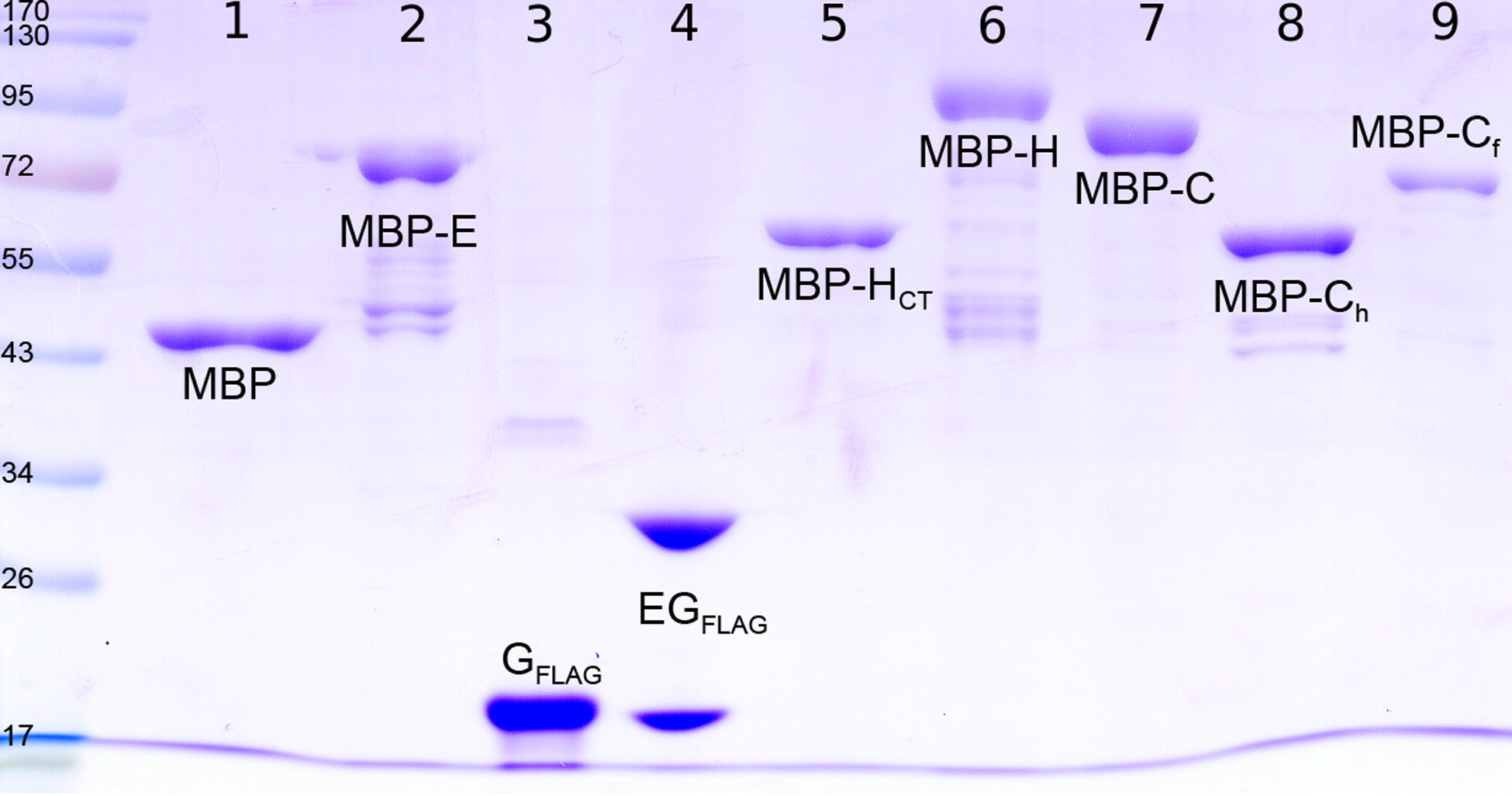

Supplement: Figure S1 — SDS-PAGE of subunit probes used in this study. Left lane, molecular mass marker; (1), MBP; (2) MBP-E; (3) FLAG-G; (4) EG-FLAG; (5) MBP-HCT; 6 MBP-H; (7) MBP-C; (8) MBP-Chead; (9) MBP-Cfoot. (TIF) [file pone.0046960.s001.tif]
